# Application of critical pathway of integrative medical service for lumbar herniated nucleus pulposus: A protocol for single-centered prospective observational study

**DOI:** 10.1097/MD.0000000000033703

**Published:** 2023-05-12

**Authors:** Joon-Won Seo, Woochul Shin, Bo-Hyung Kim, Man Kyu Choi, Sung Bum Kim, Hyungsuk Kim, Jae-Heung Cho, Won-Seok Chung

**Affiliations:** a Department of Clinical Korean Medicine, College of Korean Medicine, Graduate School, Kyung Hee University, Seoul, Korea; b Department of Korean Medicine Rehabilitation, Kyung Hee University Korean Medicine Hospital, Seoul, Korea; c Department of Clinical Pharmacology and Therapeutics, College of Medicine, Kyung Hee University, Seoul, Korea; d Department of Neurosurgery, Kyung Hee University Hospital, Kyung Hee University College of Medicine, Seoul, Korea.

**Keywords:** critical pathway, integrative medical service, lumbar herniated nucleus pulposus, prospective observational study

## Abstract

Lumbar herniated nucleus pulposus (L-HNP) is a condition in which fibroblasts escape due to degenerative changes or external forces in the intervertebral disc, causing neurological symptoms by compressing the dura mater or nerve root.

**Objectives:**

The purpose of this study is to analyze and compare the effectiveness, economic feasibility, and safety of using an integrated medical service critical pathway (CP) in L-HNP patients.

**Methods:**

This single-center prospective observational study will be performed at Kyung Hee University Medicine Hospital and Kyung Hee University Korean Medicine Hospital. The inclusion criteria are a diagnosis of L-HNP on magnetic resonance imaging or computed tomography scans, age under 80 years, a visual analog scale score of 7 or higher for either lower back pain or lower extremity pain. The included 102 participants will be classified into 6 groups (n = 17 in each group): CP application with conservative treatment; CP application with open discectomy; CP application with intrabody fusion; conservative treatment without CP application; open discectomy without CP application; and interbody fusion without CP application. We will collect data on the visual analog scale, ODI, SF-36, and EQ-5D-3L scores; number of admission days; medical staff satisfaction; patients health service satisfaction; waiting time for consultations; use of pain relievers; and CP application and completion rates.

**Conclusion:**

In future, this study is expected to serve as a basis for follow-up studies on the development and application of CPs in integrated medical services for various diseases, including lumbar herniated nucleus pulposus.

## 1. Introduction

Lumbar herniated nucleus pulposus (L-HNP) is a condition in which fibroblasts undergo degenerative changes or external forces in the intervertebral disc and a part of the nucleus escapes, causing neurological symptoms due to compression of the dura mater or nerve root.^[1]^ According to a study by the Health Insurance Review and Assessment Service in 2021, intervertebral disc disorder is the 4th largest medical inpatient disease and 9th largest contributor to Western medical expenses and the 4th largest medical inpatient disease and 3rd largest contributor to Korean medical expenses in Korean.^[2]^ In Western medicine (WM), L-HNP treatment is divided into conservative and surgical treatments. Lumbar surgery, which mainly consists of open discectomy and interbody fusion, is usually performed when conservative treatments, such as epidural blocking and facet joint blocking, are ineffective.^[3]^ In Korean medicine (KM), conservative treatment is chiefly performed and includes thermotherapy, herbal medicine, Chuna therapy, acupuncture-moxibustion therapy, and pharmaco-acupuncture therapy.^[4]^

Currently, Korea’s medical system is a dual system that separates WM and KM; however, the revision of medical laws in 2009 allowed WM doctors to cooperate with KM doctors at the hospital level. The Ministry of Health and Welfare started a Western-Korean medicine collaborative pilot project in 2016, and Step 4 of the project has been in progress since April 15, 2022.^[5]^ At the same time, the number of patients seeking integrated medical services not officially included in the existing medical system, such as diet and dance therapy, is increasing due to improved living standards, increased health interest, and increases prevalence of chronic incurable diseases.^[6]^

In addition to the existing cooperation system between WM and KM, integrated medical services provide comprehensive medical services to patients through complementary and alternative medicine (CAM), the safety and effectiveness of which have been verified in areas where conventional medicine has limitations (mainly mental, spiritual, and social health).^[7]^ Recently, the Ministry of Health and Welfare conducted an integrated medical research support project to prepare a model of integrated medical care, a new medical paradigm in which WM and KM doctors develop integrated diagnosis and treatment technologies with equal qualifications, encompassing various complementary and alternative medicine techniques.^[8]^

The critical pathway (CP) is a tool that lists the interventions of doctors, nurses, and other medical personnel over time to standardize the contents of medical interventions to be applied and maximize efficiency while minimizing medical costs within a fixed hospitalization period.^[9–11]^ After CP was first applied in the medical environment in the United States in the 1990s, it has been applied in clinical settings in Korea since the late 1990s. Recently, there has been a growing movement to introduce and apply CP in the clinical field of KM to improve the quality of medical care and patient satisfaction with medical services.

To date, CP has been studied in WM^[12,13]^ and KM^[14]^ for L-HNP, a commonly encountered disease in both WM and KM contributing to the economic burden. Although there have been studies on CP based on collaboration between WM and KM for patients with acute pain after lumbar spine surgery,^[15]^ there have been no studies on an integrated medical service CP.

An integrated medical service CP encompassing WM, KM, and CAM for L-HNP has been developed at the Department of Neurosurgery and Korean Rehabilitation Medicine at Kyung Hee Medical Center. However, further research is necessary before its clinical application.

In this study, patients with L-HNP were divided into groups that received integrated medical service CP and those that did not. They were prospectively observed to analyze and compare the effectiveness, economic feasibility, and safety of the CP.

## 2. Materials and methods

### 2.1. Study design

This single-center prospective observational study will be performed at Kyung Hee University Medicine Hospital and Kyung Hee University Korean Medicine Hospital to analyze the effectiveness, economy, and safety of using an integrated medical service CP in patients with L-HNP. The patients will be divided into groups that apply critical pathway of integrative medical services and those who do not.

From among the patients who agree to participate in the study, those who meet the inclusion criteria will be registered as research subjects. Among them, those who agree to participate in the integrated medical service CP will be assigned to the test group (integrated medical service CP applied group) and those who do not will be assigned to the control group (integrated medical service CP non-applied group). Both groups are further divided into a conservative treatment (CONS) group, an open discectomy (DISCEC) group, and an interbody fusion (FUSION) group based on the main medical treatment. In the test group, inpatient treatment will be administered according to the pathway of the prescribed protocol. In the control group, inpatient treatment will be administered based on the treating doctor’s judgment, without a prescribed pathway. The test group patients will be discharged after being hospitalized for a set hospitalization date, whereas in the control group, the time of discharge will be determined according to the judgment of the clinician. The study will be completed when the patients are evaluated during an outpatient visit to the Department of Neurosurgery of Kyung Hee Medical Center 4 weeks after discharge.

### 2.2. Study registration

The trial protocol was approved by the Institutional Review Board of Kyung Hee University Korean Medicine Hospital in July, 2022 (KHUH-2022-06-024-002).

This protocol was registered with the Clinical Research Information Service (CRIS) on 02 Sep 2022 (KCT0007671; https://cris.nih.go.kr/cris/search/detailSearch.do?seq=22923&search_page=L).

### 2.3. Participants

Patients with L-HNP visiting the Neurosurgery outpatient department of Kyung Hee Medical Center judged to need inpatient treatment will be guided to this study. Doctors and research nurses will select/exclude these patients after obtaining informed consent from those who express their intention to participate in the study. The doctors and research nurses will evaluate whether the patients meet the inclusion/exclusion criteria and enroll patients deemed suitable for this study. Depending on their intention to participate in the integrated medical service CP application, participants will be assigned to the test and control groups.

#### 2.3.1. Inclusion criteria

(1). Diagnosis of L-HNP through magnetic resonance imaging or computed tomography.
(2). Ages <80 years.
(3). Complaint of both lower back pain and lower extremity pain at the same time.
(4). Visual analog scale (VAS) score of 7 or higher for either lower back pain or lower extremity pain.
(5). Voluntary agreement to participate in this study.

#### 2.3.2. Exclusion criteria

(1). Suspected malignant tumor, spinal infection, or osteoporotic compression fracture.
(2). Need of immediate surgery due to another disease.
(3). Serious underlying disease.
(4). More pain due to musculoskeletal disease than due to L-HNP.
(5). Medical benefits, Auto insurance.
(6). Other patients whom the investigator deems unsuitable to apply CP in this clinical trial.

### 2.4. Clinical trial plan and critical pathway schedule

Among patients with lumbar intervertebral disc herniation visiting the Neurosurgery outpatient department of Kyung Hee Medical Center, patients with a VAS score of 7 or higher who require hospitalization can consent to participate in this study. Following patient consent, the doctors and research nurses will screen the patients according to the inclusion and exclusion criteria, enroll those who pass the screening, and confirm the participants intention to receive treatment using an integrated medical service CP. Depending on their intention to participate in the CP application, participants will be classified into the test or control group. Upon admission, the patients will be assigned to the CONS group, DISCEC group, and FUSION groups according to the judgment of the medical staff. The test group will receive inpatient treatment according to a previously developed integrated medical service CP plan, and the control group will receive inpatient treatment according to the clinician’s judgment without a set protocol. The study will be deemed complete when all patients will be evaluated during an outpatient visit to the Neurosurgery Department of Kyung Hee Medical Center 4 weeks after discharge.

#### 2.4.1. CP applied group

**Figure 1.**
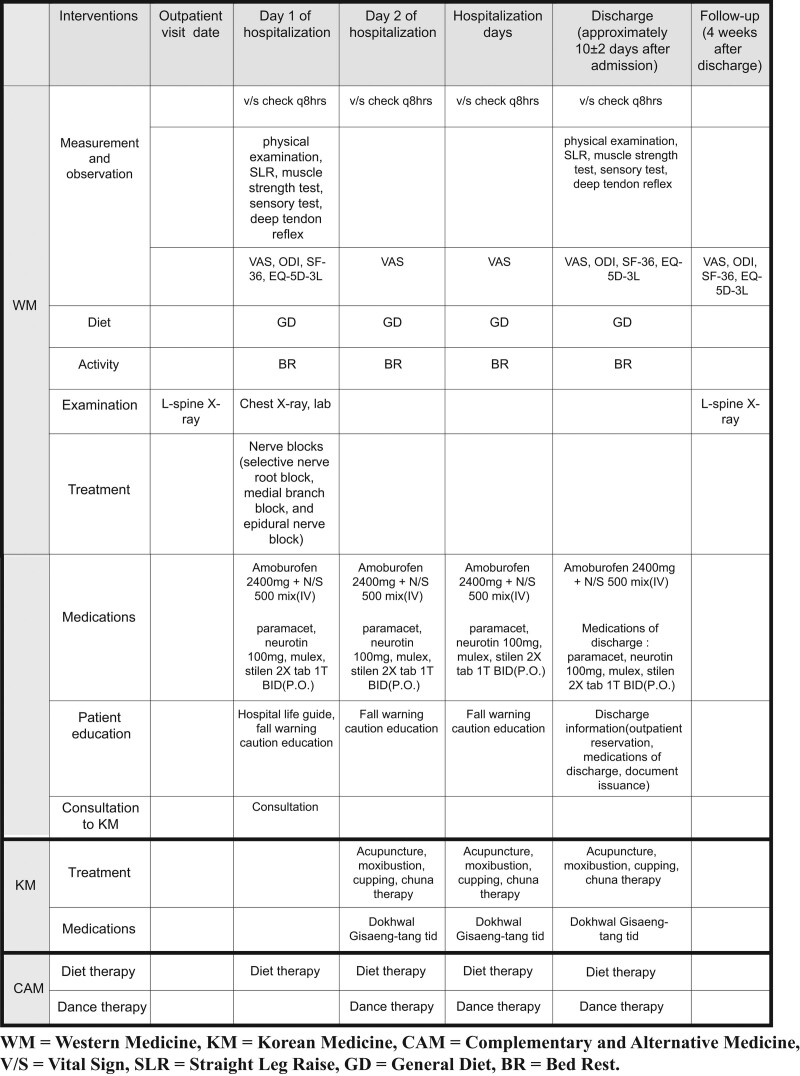
Critical pathway – conservative treatment group (CP-CONS group). CONS = conservative treatment, CP = critical pathway.

**Figure 2.**
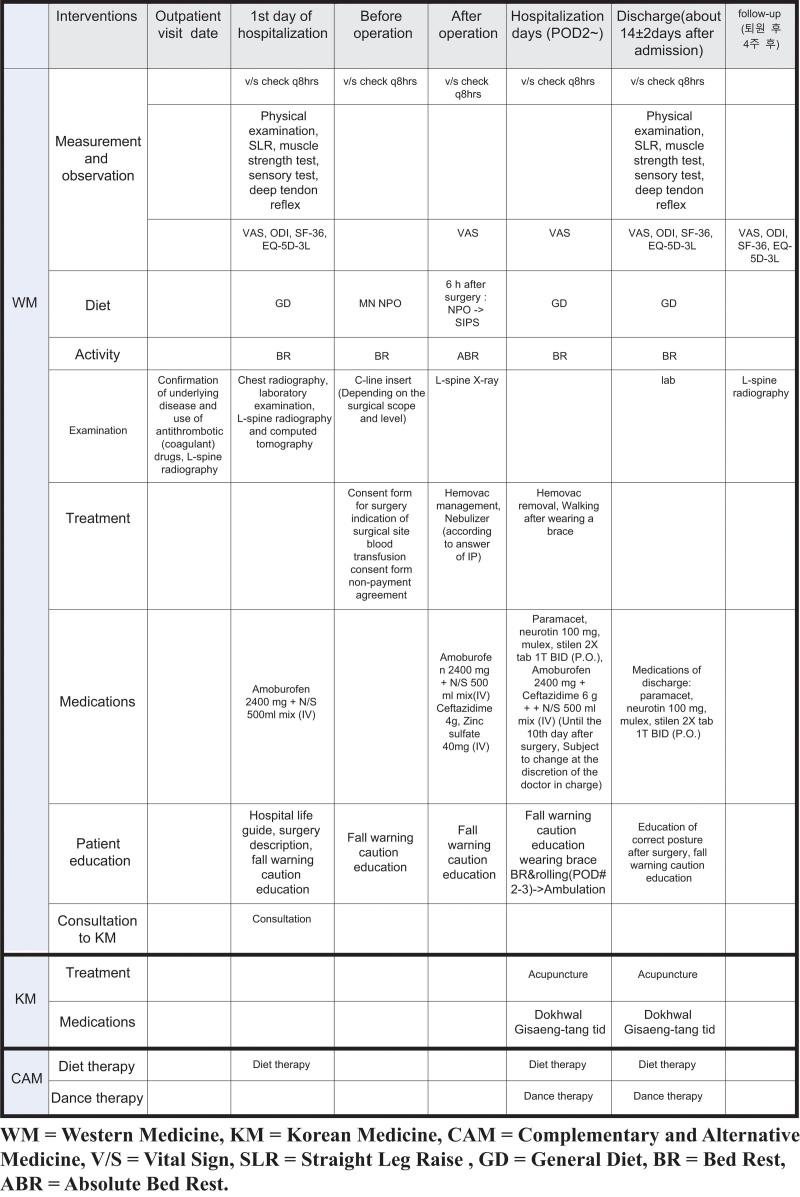
Critical pathway – open discectomy group (CP-DISCEC group). CP = critical pathway, DISCEC = open discectomy.

**Figure 3.**
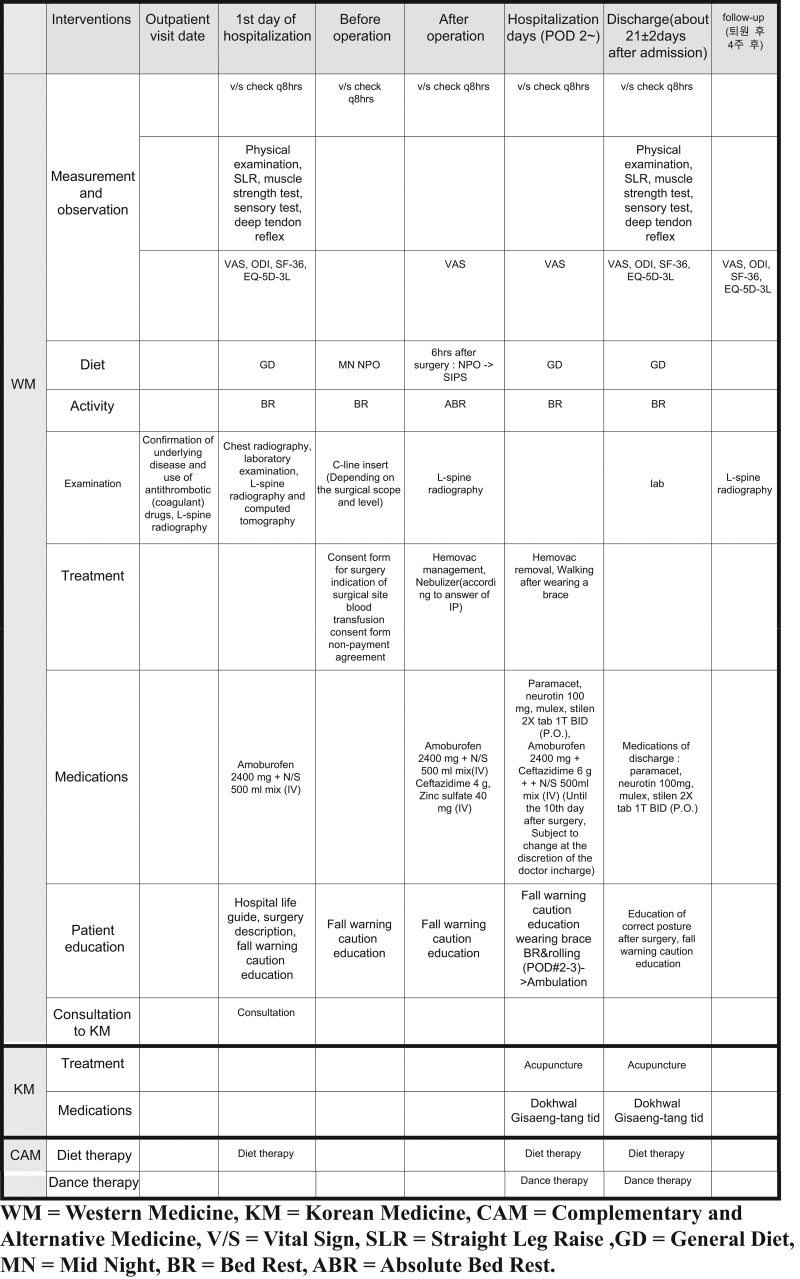
Critical pathway – interbody fusion group (CP-FUSION group). CP = critical pathway, FUSION = interbody fusion.

The CP-CONS group will follow the treatment schedule presented in Figure 1, the CP-DISCEC group will follow the treatment schedule shown in Figure 2, and the CP-FUSION group will follow the treatment schedule shown in Figure 3.

#### 2.4.2. CP non-applied group

According to the medical treatment method, the patients will be classified into the control-CONS group, control-DISCEC group, and control-FUSION group. In addition to WM treatment, KM treatment, such as acupuncture, moxibustion, cupping treatment, Chuna therapy, and herbal medicine treatment may be administered as necessary.

### 2.5. Sample size

A previous study^[16]^ evaluated the clinical effect of warm acupuncture on patients with lumbar disc herniation. The treatment group received warm acupuncture, the control group received traction treatment, and both groups were treated twice. The mean and standard deviation (mean ± SD) of the patients VAS scores before and after treatment are shown in the table 1.

**Table 1:** The mean and standard deviation (mean ± SD) of the patients visual analogue scale scores before and after treatment.

	Subjects	Before treatment (B)	After treatment (A)
Treatment group	45	6.14 ± 1.29	2.27 ± 0.84
Control group	37	6.09 ± 1.21	3.37 ± 0.93

SD = standard deviation.

#### 2.5.1. Average change in the VAS score

The average change in VAS was calculated as 2.27 to 6.14 = −3.87 in the treatment group and 3.37 to 6.09 = −2.72 in the control group. Therefore, the difference in the change in the VAS score before and after treatment between the 2 groups was d=−3.87 to (−2.72) = −1.15.

#### 2.5.2. Variance in the change in the VAS score

Assuming that the correlation coefficient (ρ) for pre- and posttreatment was 0.5, the variance in the VAS score change before and after treatment for the treatment and control groups was calculated as follows:

Treatment group: Var(A−B)=Var(A)+Var(B)−2ρSD(A)SD(B)$=1.29^{2}+0.84^{2}-2*0.5*1.29*0.84\approx 1.13^{2}$

Control group: Var(A−B)=Var(A)+Var(B)−2ρSD(A)SD(B)$=1.21^{2}+0.93^{2}-2*0.5*1.21*0.93\approx 1.10^{2}$

#### 2.5.3. Pooled variance calculation

The pooled variance was calculated to determine the number of subjects, assuming that the variance of the VAS score change before and after treatment in the treatment and control groups was the same.


$$\begin{array}{l} \sigma^{2}=\frac{\left(45-1\right)\times 1.13^{2}+\left(37-1\right)\times 1.10^{2}}{46+37-2}\approx 1.12^{2} \end{array}$$

#### 2.5.4. Calculation of the number of subjects

Assuming that the significance level (α) is 5% and the test power (1-β) is 80%, the number of subjects in each group is calculated so that the ratio of the 2 groups is 1:1.


$$\begin{array}{l} n=\frac{2\sigma^{2}{\left(Z_{1-\beta }+Z_{1-\frac{\alpha }{2}}\right)}^{2}}{d^{2}}=\frac{2\times 1.12^{2}{\left(Z_{0.8}+Z_{0.975}\right)}^{2}}{{\left(-1.15\right)}^{2}}\approx 15 \end{array}$$

The CP group is divided into the CONS, DISCEC, and FUSION groups, and CP non-applied group is also divided into the corresponding 3 groups. The minimum number of subjects required for this study is 15 per group, with a total of 90; however, considering the dropout rate (10%), a total of 102 subjects (17 per group) will be enrolled.

### 2.6. Intervention

#### 2.6.1. Medical intervention

##### 2.6.1.1. CONS group

Depending on the patient’s condition, the doctor will select and perform one of the following 3 nerve block procedures: selective nerve root, medial branch, and epidural nerve blocks.

##### 2.6.1.2. DISCEC group

The surgeon will surgically decompress the nerve by removing the protruding intervertebral disc compressing the nerve root after excising the lumbar nerve root and partially resecting the lumbar lamina after incising the patient’s skin.

##### 2.6.1.3. FUSION group

Doctors use the interbody fusion method to surgically resolve spinal instability that may occur in the future when extensive posterior arch and facet joints are included during nerve decompression. The approach can be classified as anterior, lateral, and posterior fusion.

##### 2.6.1.4. Medication

• Intravenous infusion of amoburofen + normal saline 500 mL mix, flumarin 1000 mg bid.
• Per oral administration of Paramacet 100 mg bid, neurontin 100 mg bid, Mulex Tab bid, and Stillen 2X Tab bid.

#### 2.6.2. Korean medical intervention

##### 2.6.2.1. Acupuncture treatment

A KM doctor will insert a needle into an acupuncture point suitable for back and lower extremity pain depending on the patient’s condition. The immersion time will be 15 minutes. To ensure safety, when performing acupuncture near the surgical site postoperatively, the presence or absence of a hematoma will be first confirmed through ultrasonography.

##### 2.6.2.2. Moxibustion treatment

A KM doctor will administer moxibustion treatment on acupuncture points suitable for back and lower extremity pain depending on the patient’s condition.

##### 2.6.2.3. Cupping therapy

A KM doctor will perform cupping therapy on acupuncture points suitable for back and lower extremity pain depending on the patient’s condition. The immersion time will be 5 minutes.

##### 2.6.2.4. Chuna treatment

A KM doctor will perform the Chuna technique for suitable patients with lumbar disc herniation according to the patient’s condition.

##### 2.6.2.5. Herbal medication

The conservative and surgical treatment groups will take Dokhwal Gisaeng-tang tid.

#### 2.6.3. Complementary and alternative medical intervention

##### 2.6.3.1. Diet therapy

Clinical nutritionists will check the patient’s underlying disease and current physical condition through consultation and prescribe a suitable diet.

##### 2.6.3.2. Dance therapy

A customized dance therapy program with different types of dances can be developed in person or remotely depending on the hospital conditions during the COVID-19 pandemic.

### 2.7. Outcomes

#### 2.7.1. Efficacy evaluation

##### 2.7.1.1. Primary outcome

Considering the CONS, DISCEC, and FUSION groups, we will evaluate whether the decrease in the VAS score in the CP group is statistically significant compared to that in the corresponding control group on day 1 of hospitalization and 4 weeks after discharge.

##### 2.7.1.2. Secondary outcomes

1. For the CONS, DISCEC, and FUSION groups, we will evaluate whether the decrease in the VAS score of the CP group is statistically significant compared to that in the corresponding control group on day 1 of hospitalization and discharge.
2. For the 3 treatment groups, we will evaluate whether the changes in the ODI, SF-36, and EQ-5D-3L scores in the CP group are statistically significant compared to those in the corresponding control group on day 1 of hospitalization and 4 weeks after discharge.
3. For the 3 treatment groups, we will evaluate whether the changes in the ODI, SF-36, and EQ-5D-3L scores in the CP group are statistically significant compared to those in the corresponding control group on day 1 of admission and discharge.
4. The average length of hospitalization will be compared between the CP group and the corresponding control group for all 3 treatment groups.
5. After the patient is discharged from the hospital, a questionnaire for employee satisfaction will be used to evaluate the satisfaction of doctors, nurses, and hospital staff with the application of CP.
6. After the patient is discharged from the hospital, the patient’s satisfaction with the medical services will be investigated through a questionnaire, and the satisfaction scores between the CP group and the corresponding control group will be compared.
7. We will compare the consultation waiting time between the CP group and the corresponding control group.
8. In addition to the fixed oral preparations and intravenous infusions, the amount of pro re nate (Pro Re Nate) analgesic used for pain will be investigated and compared between the CP group and the corresponding control group.
9. The CP application rate and completion rate will be analyzed in the 3 groups.

#### 2.7.2. Economic feasibilty evaluation

##### 2.7.2.1. Cost-utility analysis

Using the EQ-5D score measured 4 weeks after discharge, we will compare the QALY value in each treatment group between the CP applied group and the CP non-applied group, and the medical expense in each group until 4 weeks after discharge will be calculated. We will conduct a cost-effectiveness analysis by calculating the ICER based on the difference in costs between the groups and the QALY value.

##### 2.7.2.2. Cost-effectiveness analysis

The VAS score will be measured 4 weeks after discharge to determine the reduction in the VAS score in each CP applied and non-applied group, and the medical expense in each group until 4 weeks after discharge will be calculated. A cost-effectiveness analysis will be performed by calculating the ICER based on the difference in costs between the groups and the reduction in the VAS score.

#### 2.7.3. Safety evaluation

##### 2.7.3.1. Adverse events

Adverse events will be determined based on the severity and causal relationship with the test drug, and the incidence rate of adverse events, incidence rate of adverse events that caused dropout, and expression rate of serious adverse events will be analyzed.

##### 2.7.3.2. Clinical pathological examinations (hematologic/hematochemical test)

The difference in the pathological findings at the time of hospitalization and discharge will be tested using a paired *t* test for continuous variables, and the differences between the groups will be evaluated using a *t* test.

##### 2.7.3.3. Vital signs (blood pressure, pulse, respiratory rate, and body temperature)

The vital signs will be evaluated 3 times a day at 8-hour intervals from admission.

### 2.8. Statistical analysis

#### 2.8.1. Efficacy evaluation analysis

##### 2.8.1.1. Primary outcome analysis

The primary outcomes will be analyzed based on the treatment groups. For each group, a normal distribution will be assumed for the difference in the VAS score measured on day 1 of hospitalization and at 4 weeks after discharge, and a *t* test will be performed. The means between the treatment groups (CP and control groups) will be compared. If normality is confirmed through the normality test for the VAS scores, the Mann–Whitney test will be performed.

##### 2.8.1.2. Secondary outcome analysis

1. The differences in VAS scores measured on day 1 of hospitalization and at 4 weeks after discharge in each treatment group will be analyzed with a linear model assuming a normal distribution. In the analysis model, including the treatment group (CP and control groups), age, sex, type of insurance related to back pain, region of residence, Charlson Comorbidity Index (CCI), surgery status, and hospitalization record as covariates and factors, we will evaluate if the VAS score reduction in the CP group is statistically significant compared to that in the control group. If the normality of the VAS scores is not satisfied, we will use a sandwich estimator or perform a nonparametric statistical analysis.
2. The differences in VAS scores measured on the day of hospitalization and discharge in each treatment group will be analyzed with a linear model assuming a normal distribution. In the analysis model, including the treatment group (CP and control groups), age, sex, type of insurance related to back pain, region of residence, CCI, surgery status, and hospitalization record as covariates and factors, we will evaluate if the VAS score reduction in the CP group is statistically significant compared to that in the control group. If normality of the VAS scores is not satisfied, we will use a sandwich estimator or perform a nonparametric statistical analysis.
3. The differences in ODI, SF-36, and EQ-5D-3L scores measured on day 1 of hospitalization and 4 weeks after discharge in each treatment group will be analyzed with a linear model assuming a normal distribution. In the analysis model, including the treatment group (CP and control groups), age, sex, type of insurance related to back pain, region of residence, CCI, surgery status, and hospitalization record as covariates and factors, we will evaluate if the VAS score reduction in the CP group is statistically significant compared to that in the control group. If normality of the ODI, SF-36, and EQ-5D-3L scores is not satisfied, we will use a sandwich estimator or perform a nonparametric statistical analysis.
4. The differences in ODI, SF-36, and EQ-5D-3L scores measured on the day of hospitalization and discharge in each treatment group will be analyzed with a linear model assuming a normal distribution. In the analysis model, including the treatment group (CP and control groups), age, sex, type of insurance related to back pain, region of residence, CCI, surgery status, and hospitalization record as covariates and factors, we will evaluate if the VAS score reduction in the CP group is statistically significant compared to that in the control group. If normality of the ODI, SF-36, and EQ-5D-3L scores is not satisfied, we will use a sandwich estimator or perform a nonparametric statistical analysis.
5. The average length of hospital stay in the CP group and control group will be compared by a 2-sample *t* test. If normality is not satisfied, the Wilcoxon rank-sum test will be performed.
6. After patient discharge, we will perform the χ^2^ test or Fisher exact test for the test and control groups (CP applied and CP non-applied group) for staff satisfaction data obtained through a questionnaire.
7. After patient discharge, we will perform a homogeneity test for the test and control groups (CP applied and CP non-applied groups) to evaluate patient satisfaction using the data obtained from the questionnaires.
8. The consultation waiting time will be compared between the CP and control groups was compared using a 2-sample *t* test. If normality is not satisfied, the Wilcoxon rank-sum test will be performed.
9. To compare the additional amount of pro re nate analgesic consumed between the CP and control groups, a 2-sample *t* test will be used. If normality is not satisfied in the normality test for additional analgesic use, the Wilcoxon rank-sum test will be performed.
10. We will perform ANOVA (analysis of variance) to compare the average CP application and completion rates in the CONS, DISCEC, and FUSION groups of the CP group.

#### 2.8.2. Safety evaluation analysis

Safety evaluation variables, including clinical laboratory test results, will be presented descriptively. However, significantly abnormal test results may be described individually if necessary.

##### 2.8.2.1. Adverse events

All adverse events occurring in this clinical study will be standardized by system organ class (SOC) and preferred term (PT) based on the MedDRA dictionary. For adverse events and adverse drug events occurring in clinical research trials, the frequency (number of subjects), percentage, and number of occurrences are presented based on the SOC and PT according to the CP applied and CP non-applied groups. For adverse reactions that occurred by severity, Based on SOC and PT, the frequency of occurrence (number of subjects), percentage, and number of occurrences are presented.

For serious adverse reactions, the frequency (number of subjects), percentage, and number of occurrences will be presented based on the SOC and PT according to the CP applied and CP non-applied groups. The adverse reactions, duration, course, severity, outcome, and causal relationships will be summarized in a list. In addition, individual lists of all subjects with adverse events will be presented, including the group, reported adverse events, start date/time and end date/time, course, severity, seriousness and relationship, actions taken, and final outcome. Adverse events leading to discontinuation of the study will be listed separately. The adverse events will be summarized based on the preferred terms according to MedDRA.

##### 2.8.2.2. Vital signs and physical examination

Values measured at each time point and changes from the pre-dose test results will be summarized with descriptive statistics (number (N), median, mean, SD, minimum, and maximum values).

##### 2.2.8.3. Clinical pathology test

For quantitative variables (e.g., complete blood count and clinical chemistry), descriptive statistics will be summarized (N, median, mean, SD, minimum, and maximum values). For qualitative variables, the test results before and after administration will be summarized and described. In addition, the differences in the laboratory findings at the time of hospitalization and at discharge will be tested using a paired *t* test for continuous variables, and the groups will be compared using a *t* test.

## 3. Discussion

As part of the integrated medical support project, a critical pathway (CP) of integrated medical services that combines WM, KM, and CAM treatments for herniated discs of the lumbar spine, a common inpatient disease with high medical costs, was developed. In this study, the effectiveness, economic feasibility, and safety of the CP for inpatients in actual clinical settings will be prospectively analyzed by dividing the patients into groups that did and did not undergo treatment with the integrated medical service CP. This study is expected to serve as a stepping stone for further research on the development and application of this integrated medical service CP for various diseases, including lumbar disc herniation.

## Acknowledgments

We would like to thank Editage (www.editage.co.kr) for English language editing.

## Author contributions

**Conceptualization:** Joon-Won Seo, Woochul Shin, Hyungsuk Kim, Won Seok Chung.

**Data curation:** Joon-Won Seo, Woochul Shin.

**Formal analysis:** Joon-Won Seo, Woochul Shin.

**Funding acquisition:** Won Seok Chung.

**Investigation:** Joon-Won Seo, Woochul Shin, Bo-Hyung Kim, Man Kyu Choi, Sung Bum Kim, Hyungsuk Kim, Jae-Heung Cho, Won Seok Chung.

**Methodology:** Joon-Won Seo, Woochul Shin, Bo-Hyung Kim, Man Kyu Choi, Sung Bum Kim, Hyungsuk Kim, Jae-Heung Cho, Won Seok Chung.

**Project administration:** Joon-Won Seo, Woochul Shin, Bo-Hyung Kim, Man Kyu Choi, Sung Bum Kim, Hyungsuk Kim, Jae-Heung Cho, Won Seok Chung.

**Resources:** Joon-Won Seo, Woochul Shin, Bo-Hyung Kim, Man Kyu Choi, Sung Bum Kim, Hyungsuk Kim, Jae-Heung Cho, Won Seok Chung.

**Software:** Joon-Won Seo, Woochul Shin, Bo-Hyung Kim, Man Kyu Choi, Sung Bum Kim, Hyungsuk Kim, Jae-Heung Cho, Won Seok Chung.

**Supervision:** Joon-Won Seo, Woochul Shin, Bo-Hyung Kim, Man Kyu Choi, Sung Bum Kim, Hyungsuk Kim, Jae-Heung Cho, Won Seok Chung.

**Validation:** Joon-Won Seo, Woochul Shin, Sung Bum Kim, Hyungsuk Kim, Won Seok Chung.

**Visualization:** Joon-Won Seo, Woochul Shin.

**Writing – original draft:** Joon-Won Seo, Woochul Shin.

**Writing – review & editing:** Sung Bum Kim, Hyungsuk Kim, Won Seok Chung.
